# Hypomagnesemia and incident delirium in hospitalized older persons

**DOI:** 10.1007/s40520-023-02357-3

**Published:** 2023-01-28

**Authors:** Virginia Boccardi, Sara Ercolani, Rocco Serra, Valentina Bubba, Alessandro Piccolo, Michela Scamosci, Alfredo Villa, Carmelinda Ruggiero, Patrizia Mecocci

**Affiliations:** 1 Institute of Gerontology and Geriatrics, Department of Medicine and Surgery, Santa Maria della Misericordia Hospital, University of Perugia, Piazzale Gambuli 1, 06132 Perugia, Italy; 2 Department of Clinical Pathology, Santa Maria della Misericordia Hospital, Perugia, Italy; 3 Division of Clinical Geriatrics, NVS Department, Karolinska Institutet, Stockholm, Sweden

**Keywords:** Magnesium, Older, Geriatrics, Delirium, Nutrition

## Abstract

**Background:**

Altered serum magnesium (Mg) levels in older persons have been hypothesized to have a role in predicting hospitalization and mortality. Hypomagnesemia and delirium are frequent problems in older patients, but no study has evaluated such an association in acute geriatric setting.

**Aims:**

We investigated the impact of hypomagnesemia on the incidence of delirium in an acute geriatric setting.

**Methods:**

This retrospective study was conducted on 209 older hospitalized patients. All subjects underwent a comprehensive geriatric assessment. Mg was measured in serum by routine laboratory methods. The presence of incident delirium was determined by the 4AT screening tool. A logistic regression model was used to assess the association between serum Mg and delirium controlling for multiple covariates.

**Results:**

209 patients (77.9% women) were included in the study. The mean age of the participants was 85.7 ± 6.50 years (range 65–100). 27 subjects (12.9%) developed delirium during the hospitalization, with no difference between genders. Subjects with delirium had lower serum magnesium levels than those without (1.88 ± 0.34 versus 2.04 ± 0.28; *p* = 0.009). Delirium risk was significantly higher in patients with lower serum magnesium levels (OR 5.80 95% CI 1.450–23.222; *p* = 0.013), independent of multiple covariates.

**Conclusion:**

Our data show that low serum Mg level is a good predictor of incident delirium in acute geriatric settings. Present findings have relevant implications for clinical management, highlighting the need for analyzing Mg concentration carefully. Whether Mg supplementation in patients with hypomagnesemia could lead to delirium prevention and/or control needs further investigation.

## Introduction

Delirium is a common geriatric syndrome defined as an abrupt change in cognitive functions (mainly attention) characterized by acute onset, fluctuating course, and behavioral abnormalities, which develop in association with another underlying medical condition [1]. Delirium can be described as prevalent, found on admission, or incident when it develops during the hospital stay. In geriatrics delirium may represent the signature of the uncommon presentation of many acute diseases, particularly in frail persons, and it has been recognized as an independent risk factor for increased length of hospital stay (LOS), delayed or restricted recovery of functional decline, increased risk of adverse outcomes, institutionalization, and mortality [2]. Patients with predisposing factors, such as advanced age, preexisting dementia, or underlying cognitive and functional frailty, are at high risk of delirium [3] in the presence of precipitating factors such as electrolyte abnormalities [4, 5], a very frequent condition in the elderly.

Electrolytes are essential components in the human body with the function of cell membrane potential maintenance, nerve impulse and transmission, and intra- and extracellular fluid balance preservation [6]. Aging per se impairs homeostatic mechanisms leading to a significant decline in adaptive capacity. Thus, an acute event that promotes a minimal change in circulating electrolytes in younger persons may produce higher variability in older [6, 7].

Over the past decades, among electrolytes, many studies have demonstrated the clinical relevance and biological significance of magnesium (Mg) in geriatric patients [8] and, in particular, the total body Mg deficit associated with aging [9]. Mg represents the most abundant electrolyte in the body, and it is mainly distributed in bones (about 60%) and muscles (around 20%), while only 1% of the total body Mg is available in extracellular fluids [10]. Mg is involved in different biochemical processes and reactions, including adenosine triphosphate (ATP) metabolism, muscle contraction and loosening, blood pressure regulation, neuronal activity, as well as neurotransmitter release and modulation [11]. Growing evidence is available on the involvement of Mg in many physiological processes in the whole body and across physiological functions, including endocrine, cardiac, renal, and central nervous systems [6, 9]. Even if the serum Mg as measured could not be an accurate reflection of magnesium stores, it can be used as a surrogate marker in clinical practice. In detail, it has been established an association between hypomagnesemia and some clinical neurological manifestations, including depression, stroke, migraine, and cognitive dysfunctions [12]. Although it is reasonable to postulate a similar relationship between hypomagnesemia and delirium, only a study has recently examined this possible association [13]. The study has been performed on critically ill adults and demonstrated that hypomagnesemia increases the risk of delirium by more than two-fold compared to patients with normal Mg levels [13]. However, no evidence is available in hospitalized very old persons. Therefore, we investigated the impact of hypomagnesemia on the incidence of delirium in an acute geriatric setting.

## Materials and methods

### Subjects and study design

This is a retrospective study conducted among 860 older adults consecutively admitted to the geriatric acute care unit of Santa Maria della Misericordia Hospital (University Hospital of Perugia) from January 2017 to January 2018. Only subjects aged 65 years and more with available measurement of magnesium at admission were included, leading to a final cohort of 209 patients. Critically ill patients, subjects with terminal cancer or under chemotherapeutic drugs, subjects with a story of alcohol use or laxative abuse, and subjects with prevalent delirium were ruled out. Data on demographics, anthropometrics, physical examination, and clinical and biochemical characteristics were gathered from the hospitalization chart. The ethical committee of the University Hospital of Perugia approved the study protocol.

### Analytical method

Blood samples were collected at the entry into the hospital ward. Albumin, glycemia, calcium, phosphorous, sodium, potassium, magnesium, and creatinine were determined in serum by routine laboratory methods (Roche Diagnostics, GmbH, Mannheim, Germany). Clearance creatinine has been calculated by the BIS-1 (Berlin Initiative Study) formula and expressed as mL/min/1.73 m2 [14]. Upon our laboratory reference, hypomagnesemia was defined as a serum magnesium concentration lower than 1.8 mg/dL. Thus, the population sample was stratified into two groups: hypomagnesemia and normomagnesemia-with Mg serum levels ranging from 1.8 mg/dl to 2.6 mg/dl.

### Cognitive, functional, and nutritional assessment

Cognitive performance was evaluated, upon admission, with the Mini Mental State Examination (MMSE) as a test of general cognition [15]. To avoid the underestimation of a self-rated level of functional capacity, an informant-based rating of functional status was carried out using the Basic Activities of Daily Living (BADL) [16] and the Instrumental Activities of Daily Living (IADL) scales [17]. BADL includes six activities: bathing, dressing, toileting, transferring, continence, and feeding. IADL includes eight activities: using the telephone, shopping, meal preparation, housekeeping, laundry, use of transportation, self-administration of drugs, and handling finances. Any dysfunction in the performances of these activities was recorded as dependent on the correspondent item. Because IADL items are often gender-specific, we used the version of the scale tested for male subjects that included only five items, with housekeeping, cooking, and doing laundry excluded. The BADL score ranges from 6 (total independence) to 0 (total dependence), and IADL from 8 (in women) or 5 (in men) (total independence) to 0 (total dependence). The nutritional status was evaluated by the administration of the Mini Nutritional Assessment (MNA) [18], an instrument developed to detect malnutrition in elderly subjects.

### Delirium assessment

Trained bedside duty nurses monitored each patient’s level of sedation and agitation, and the patient was screened for delirium once per day using the 4AT tool. The 4AT is now one of the most commonly used tools in practice worldwide. The 4AT is a validated brief delirium assessment tool designed for clinical use [19]. It is scored between 0 and 12 points. We analyzed the 4AT assessment every day after admission, using the standard cutoffs of 4AT 0 (normal test), 4AT 1–3 (possible cognitive impairment, no delirium), and 4AT ≥ 4 (possible delirium ± cognitive impairment).

### Statistical analyses

The observed data were normally distributed (Shapiro‒Wilk W-test) and are presented as means ± standard deviation (SD). To assess differences among groups, unpaired *t*-test, ANOVA, or Pearson’s Chi-squared (*χ*2) test were used, as appropriate. The independent effect of serum Mg levels on delirium risk (dependent variable) was tested by a binary logistic regression analysis controlled by multiple covariates. The sample size calculation was estimated by GPower 3.1.7 software (http://www.softpedia.com). The total sample size of 209 subjects, estimated post hoc according to a global effect size *f*^2^ of 0.15 with type I error of 0.05, had a power of 98.9%. All p values are two-tailed, and the level of significance was set at *p* ≤ 0.05. Statistical analyses were performed using the SPSS 20 software package (SPSS, Inc., Chicago, IL, USA).

## Results

209 (163F/46M) patients with available Mg measurement at hospitalization were included in the study. Table 1 reports all population sample characteristics. Patients were primarily oldest-old, with a mean age of 85.7 ± 6.50 years (range 65–100), and at risk of malnutrition. No correlation was found between age and Mg levels (*r* = 0.001, *p* = 0.989), and no difference in Mg levels was found between genders (*t* = − 0.235, *p* = 0.815). Stratifying all population according to baseline serum Mg level 49 (23.4%) patients were classified in the hypomagnesemia (< 1.8 ng/dl) group and showed significantly lower serum levels of phosphorus, as well as higher use of thiazide diuretics, as compared with subjects with normomagnesemia (Table 2). No other differences were found between groups. No correlation was found between Mg levels and MNA (*r* = 0.095, *p* = 0.264), the number of used drugs (*r* = − 0.111, *p* = 0.125), or length of hospital stay (*r* = − 0.069, *p* = 0.342). Among other drugs affecting Mg plasma concentration, no difference was found in Mg levels between users and non-users of proton pump inhibitors (*t* = 0.959, *p* = 0.339) or loop diuretics (*t* = 0.993, *p* = 0.330).

**Table 1:** Baseline population sample characteristics (*n* = 209)

	Total Sample *N* = 209
Age, years	86.78 ± 6.50
Gender (female), n (%)	163 (77.9)
SBP (mmHg)	133 ± 14
DBP (mmHg)	74 ± 9
Drugs, n	6.75 ± 3.27
MMSE	23.35 ± 4.55
BADL	2.89 ± 2.17
IADL	2.38 ± 2.78
MNA	21.04 ± 3.66
Glycaemia (mg/dl)	114.03 ± 41.72
Albumin (g/dL)	3.50 ± 0.88
Calcium, corrected (mg/dL)	8.65 ± 1.63
Phosphorus (mg/dL)	3.34 ± 1.42
Magnesium (mg/dL)	2.02 ± 0.29
Potassium (mmol/L)	4.11 ± 0.55
Sodium (mmol/L)	139.94 ± 5.21
Clearance creatinine (BIS1)	39.13 ± 19.41
Length of hospital stay (days)	11.57 ± 8.11

Unless otherwise noted, data are presented as means ± standard deviation (SD)

*SBP* systolic blood pressure, *DBP* diastolic blood pressure, *MMSE* mini mental state examination (corrected by age and education), *BADL* basic activities of daily living, *IADL* instrumental activities of daily living, *MNA* mini nutritional assessment, Clearance creatinine calculated by BIS-1 (Berlin Initiative Study) formula and expressed as ml/min/1.73 m^2^.

**Table 2:** Baseline population sample characteristics (*n* = 209) stratified according to serum magnesium levels

	Hypomagnesemia *N* = 49	Normomagnesemia *N* = 160	*p*
Age, years	87.02 ± 6.88	85.40 ± 6.36	0.127
Gender (female), n (%)	39 (79.5)	124 (77.5)	0.463^a^
SBP (mmHg)	133 ± 12	134 ± 14	0.952
DBP (mmHg)	74 ± 8	72 ± 9	0.857
Drugs, n	6.55 ± 3.29	6.81 ± 3.28	0.631
MMSE	23.73 ± 6.18	23.25 ± 4.13	0.759
BADL	2.57 ± 2.25	2.99 ± 2.14	0.008
IADL	2.02 ± 2.79	2.49 ± 2.78	0.243
MNA	21.04 ± 3.66	19.92 ± 3.70	0.316
Glycaemia (mg/dl)	111.69 ± 44.25	114.74 ± 41.06	0.679
Albumin (g/dL)	3.29 ± 0.62	3.56 ± 0.94	0.061
Calcium, corrected (mg/dL)	8.29 ± 1.75	8.76 ± 1.59	0.079
Phosphorus (mg/dL)	2.87 ± 1.69	3.49 ± 1.29	0.008
Potassium (mmol/L)	4.14 ± 0.65	4.10 ± 052	0.662
Sodium (mmol/L)	139.94 ± 5.21	140.44 ± 4.98	0.300
Clearance creatinine (BIS1)	41.09 ± 21.12	38.55 ± 18.90	0.427
Length of hospital stay (days)	13.02 ± 9.79	11.13 ± 7.50	0.154
Loop diuretic use, n (%)	19 (38.7)	30 (18.7)	0.500^b^
Thiazide diuretic use, n (%)	12 (24.4)	18 (11.2)	0.022^c^
PPI use, n (%)	25 (51.0)	79 (49.3)	0.485^d^

Unless otherwise noted, data are presented as means ± standard deviation (SD)

*SBP* systolic blood pressure, *DBP* diastolic blood pressure, *MMSE* mini mental state examination (corrected by age and education, *BADL* basic activities of daily living, *IADL* instrumental activities of daily living, *MNA* mini nutritional assessment, Clearance creatinine calculated by BIS-1 (Berlin Initiative Study) formula and expressed as ml/min/1.73 m^2^, PPI: proton-pump inhibitors

a: *χ*2 = 0.096

b: *χ*2 = 0.260

c: *χ*2 = 5.349

d: *χ*2 = 0.410

In all samples, 27 (12.9%) subjects developed delirium during hospitalization with no difference between genders (*χ*^2^ = 0.935, *p* = 0.242). Table 3 reports the population’s characteristics stratified into two groups according to delirium incidence. Subjects with delirium were more cognitively compromised as well as more disabled in basic and instrumental activities of daily living. Subjects with delirium had significantly lower magnesium (1.88 ± 0.34 versus 2.04 ± 0.28; *p* = 0.009) and phosphorus (2.47 ± 1.90 versus 3.47 ± 1.29; *p* = 0.001) serum levels as compared with subjects without incident delirium. No other difference was found between groups. The incidence of delirium during hospitalization significantly differed between the hypo- and normomagnesemia groups. Among subjects with hypomagnesemia (*n* = 49), 14 (25.5%) faced delirium as compared with 13 (8.12%) in the group with a normal value of magnesium (*χ*2 = 13.940, *p* = 0.001).

**Table 3:** Baseline population sample characteristics (*n* = 209) stratified  according to delirium presence

	Delirium *N* = 27	No delirium *N* = 182	*p*
Age, years	87.15 ± 5.26	85.58 ± 6.66	0.243
SBP (mmHg)	134 ± 14	133 ± 12	0.948
DBP (mmHg)	74 ± 8	73 ± 8	0.748
Drugs, n	6.77 ± 3.40	6.74 ± 3.26	0.969
MMSE	19.63 ± 7.24	23.98 ± 3.67	0.011
BADL	1.85 ± 1.75	3.05 ± 2.19	0.008
IADL	1.35 ± 2.54	2.54 ± 2.79	0.041
MNA	19.92 ± 3.70	21.15 ± 3.65	0.252
Glycaemia (mg/dL)	119.59 ± 47.73	113.25 ± 40.92	0.506
Albumin (g/dL)	3.63 ± 0.68	3.48 ± 0.91	0.427
Calcium, corrected (mg/dL)	8.52 ± 2.01	8.66 ± 1.58	0.666
Phosphorus (mg/dL)	2.47 ± 1.90	3.47 ± 1.29	0.001
Magnesium (mg/dL)	1.88 ± 0.34	2.04 ± 0.28	0.009
Potassium (mmol/L)	4.26 ± 0.45	4.09 ± 0.56	0.691
Sodium (mmol/L)	140.44 ± 4.98	139.87 ± 5.25	0.593
Clearance creatinine (BIS1)	42.28 ± 22.85	38.66 ± 18.87	0.367
Length of hospital stay (days)	11.37 ± 8.24	11.60 ± 8.11	0.889

Data are presented as means ± SD

*MMSE* mini mental state examination (corrected by age and education), *BADL* basic activities of daily living, *IADL* instrumental activities of daily living, *MNA* mini nutritional assessment, Clearance creatinine calculated by BIS-1 (Berlin Initiative Study) formula and expressed as ml/min/1.73 m^2^.

**Table 4:** Binary logistic analysis to assess whether hypomagnesemia is associated with incident delirium controlling for multiple confounding factors (*n* = 209)

	B	OR	95% CI for OR	*p*
Age	0.051	1.052	0.941–1.117	0.372
Gender	0.186	1.204	0.253–5.732	0.815
MMSE	− 0.111	0.895	0.794–1.008	0.067
Albumin	0.028	1.029	0.359–2.950	0.958
Phosphorus	− 0.333	0.717	0.466–1.103	0.130
Hypomagnesemia	1.758	5.802	1.450–23.222	0.013
Clearance creatinine	− 0.012	0.988	0.952–1.025	0.523
Diuretics use	− 1.279	0.278	0.025–3.082	0.293

Gender indicated as M = 1 and F = 0; Clearance creatinine calculated by BIS-1 (Berlin Initiative Study) formula and expressed as mL/min/1.73 m^2^; Hypomagnesemia indicated as 1; Diuretics use stands for thiazide diuretics use. *B*: regression coefficient; *OR* odds ratio, *CI* confidence interval.

A final logistic regression analysis was performed to evaluate the effects of hypomagnesemia on the likelihood that patients have delirium (Table 4). The logistic regression model was statistically significant (*χ*2 = 19.263, *p* = 0.014). The model explained 27.4% (Nagelkerke R2) of the variance in delirium incidence and correctly classified 90.3% of cases. Subjects with hypomagnesemia were 4.92 times more likely to develop incident delirium during hospitalization. Such an association resulted independent of the main covariates, including age, gender, cognitive status (by MMSE), albumin, phosphorus, renal function, and thiazide diuretic use.

## Discussion

Our results show that older patients with incident delirium during hospitalization have significantly lower serum Mg levels compared to subjects without delirium and that hypomagnesemia increases the risk of delirium by more than five-fold.

Mg is a divalent intracellular cation most present in the human cell after potassium. At the cellular level, Mg is involved in many biochemical functions, particularly in the production and utilization of energy, as adenosine triphosphate (ATP), with a key role in ionic transport processes. Along with aging the total body Mg declines, mainly due to a reduction in oral intake, physiological changes (i.e., abnormal gastrointestinal absorption or alterations in kidney function), and the influence of polypharmacotherapy (11). Our data support this notion showing that roughly 1 to 4 (23.4%) older patients have hypomagnesemia at admission.

Reduced dietary Mg intake, Mg deprivation, Mg renal loss, and subsequent hypomagnesemia are related to increased systemic inflammation and oxidative stress, as shown in previous experimental, epidemiological, and clinical studies [20, 21]. Thus, Mg insufficiency may be considered one of the mediators linking inflammation, aging, and age-related diseases [8, 11]. Recent studies support the hypothesis that Mg may have a neuroprotective effect, and it has been recently evaluated for its role in acute stroke [22]. Interestingly, Mg has been reported to reduce neuroinflammation, even if the mechanisms of these effects are still not completely understood [23]. Mounting evidence shows that Mg levels in the central nervous system may affect several biochemical processes, and it is mainly involved in cognitive functions, including cell membrane stability and integrity, response to excitatory and inhibitory stimuli, and calcium-antagonist action with neurotransmitters balance modulation [10]. Moreover, it has been shown that hypomagnesemia may lead to hyperexcitability in the central nervous system [24], as shown by electrophysiological evidence. Neuroinflammation, neurotransmitter imbalance, and hyperexcitability are part of the physiopathology of delirium [1]; thus, a link between Mg and delirium incidence cannot be ruled out.

Here, we found that patients with hypomagnesemia exhibited a five-fold higher risk of delirium than those with normomagnesemia. Our data demonstrate that subjects with delirium have lower MMSE (as an indicator of reduced cognitive performances) and BADL and IADL (as indicators of functional declines) scores, delineating a complex clinical phenotype known as “cognitive frailty”. As an indicator of cognitive frailty, a lower cognitive function may represent an important predominant factor in our acute population. In fact, delirium may be considered a signature of cognitive frailty as well as a risk factor for dementia in older populations, even if their interrelationship remains poorly understood. However, previous studies have also documented that dementia, in turn, represents the leading risk factor for delirium [3]. Again, we found that phosphorus levels are significantly lower both in subjects with delirium and hypomagnesemia. This result is not surprising, considering that alterations in Mg and phosphorus serum concentrations are frequently observed in acute patients [25]. However, the relationship between phosphorus and magnesium metabolism is still unclear [26]. It is important to recognize that some drugs frequently used in older persons can cause symptomatic hypomagnesemia. The drugs more often associated with hypomagnesemia are diuretic classes due to their mechanisms of action modulating renal tubular magnesium reabsorption and excretion. Thus, we also considered the current diuretic therapy in all subjects. We found that subjects with hypomagnesemia were more likely to use thiazide diuretics. Thus, we considered this habit as covariate. The regression analysis adjusted for multiple covariates (including age, gender, MMSE, albumin, phosphorus, renal function, and thiazide diuretic treatment) demonstrated that hypomagnesemia was independently associated with an increased risk of delirium. The model explained almost 30% of the variance in delirium incidence. A possible explanation for this finding may be the reduced neuroprotective effect in patients with hypomagnesemia. Specifically, deficiency of Mg—a vital element for establishing the electrical potential across cell membranes, activating enzymes, and regulating calcium metabolism [22]—can induce alteration in cognitive function. In this context, hypomagnesemia can precipitate delirium in frail persons. Alterations in serum concentrations of Mg are frequently observed in acute patients in emergency settings or intensive-care areas. Accordingly, only a previous study has shown the relationship between hypomagnesemia and the incidence of delirium in adult patients in intensive medical wards [13]. Our finding that hypomagnesemia is associated with an increased risk of delirium in the acute geriatric setting is important, with many clinical consequences in its identification and management. Our results could help to detect patients at risk with the play out of symptomatic strategies to manage delirium or the polypharmacotherapy deprescribing (i.e., the drug withdrawal, including diuretics) that can resolve the problem. This study has several strengths, including the comprehensive geriatric assessment. The limited number of subjects and the cross-sectional nature represent the major limitation of this study. However, considering the magnitude of the effect size found in the logistic regression analysis, the actual sample size has a strong power (over 99%). Prospective and multicenter studies are necessary to confirm our observation. Another limitation is the unavailability of all other clinical conditions that can influence Mg levels, thus further studies are needed to confirm our hypothesis. However, we included the main factors involved in Mg alterations in an acute geriatric population, as well as subjects with terminal cancer or under chemotherapeutic drugs, subjects with a story of alcohol use or laxative abuse were properly ruled out.

In conclusion, these data show that hypomagnesemia in hospitalized older subjects is a strong predictive marker of delirium. These results have relevant implications for clinical management in acute geriatric settings, highlighting the need for considering Mg concentration carefully. Whether Mg supplementation in subjects at risk could lead to delirium prevention and/or management may be viewed as an essential issue to be tested in designed randomized controlled trials.

## Data Availability

The datasets used and/or analyzed during the current study will be available from the corresponding author on reasonable request.

## References

[1] Bellelli G, Moresco R, Panina-Bordignon P (2017). Is delirium the cognitive harbinger of frailty in older adults? A review about the existing evidence. Front Med.

[2] Marcantonio ER (2017). Delirium in hospitalized older adults. N Engl J Med.

[3] Fong TG, Davis D, Growdon ME (2015). The interface between delirium and dementia in elderly adults. Lancet Neurol.

[4] Dyer CB, Ashton CM, Teasdale TA (1995). Postoperative delirium: a review of 80 primary data-collection studies. Arch Intern Med.

[5] Wang LH, Xu DJ, Wei XJ (2016). Electrolyte disorders and aging: risk factors for delirium in patients undergoing orthopedic surgeries. BMC Psychiatry.

[6] Schlanger LE, Bailey JL, Sands JM (2010). Electrolytes in the aging. Adv Chronic Kidney Dis.

[7] El-Sharkawy AM, Sahota O, Maughan RJ (2014). The pathophysiology of fluid and electrolyte balance in the older adult surgical patient. Clin Nutr.

[8] Barbagallo M, Dominguez L (2010). Magnesium and aging. Curr Pharm Des.

[9] Luckey AE, Parsa CJ (2003). Fluid and electrolytes in the aged. Arch Surg.

[10] Barbagallo M, Belvedere M, Dominguez LJ (2009). Magnesium homeostasis and aging. Magnes Res.

[11] Barbagallo M, Veronese N, Dominguez LJ (2021). Magnesium in aging, health and diseases. Nutrients.

[12] Xue W, You J, Su Y (2019). The effect of magnesium deficiency on neurological disorders: a narrative review article. Iran J Public Health.

[13] Kim J-Y, Lee HJ, Lee HY (2022). The effects of hypomagnesemia on delirium in middle-aged and older adult patients admitted to medical intensive care units. Acute Crit Care.

[14] Pottel H, Hoste L, Dubourg L (2016). An estimated glomerular filtration rate equation for the full age spectrum. Nephrol Dial Transplant.

[15] Folstein MF, Folstein SE, McHugh PR (1975). “Mini-mental state”. A practical method for grading the cognitive state of patients for the clinician. J Psychiatr Res.

[16] Katz S, Ford AB, Moskowitz RW (1963). Studies of illness in the aged: the index of ADL: a standardized measure of biological and psychosocial function. JAMA J Am Med Assoc.

[17] Lawton MP, Brody EM (1969). Assessment of older people: self-maintaining and instrumental activities of daily living. Gerontologist.

[18] Guigoz Y, Vellas B (1999). The Mini Nutritional Assessment (MNA) for grading the nutritional state of elderly patients presentation of the MNA, history and validation. Nestle Nutr Workshop Ser Clin Perform Programme.

[19] Tieges Z, Maclullich AMJ, Anand A (2021). Diagnostic accuracy of the 4AT for delirium detection in older adults: systematic review and meta-analysis. Age Ageing.

[20] Malpuech-Brugère C, Nowacki W, Daveau M (2000). Inflammatory response following acute magnesium deficiency in the rat. Biochim Biophys Acta—Mol Basis Dis.

[21] Bussière FI, Tridon A, Zimowska W (2003). Increase in complement component C3 is an early response to experimental magnesium deficiency in rats. Life Sci.

[22] Saver JL, Starkman S, Eckstein M (2015). Prehospital use of magnesium Sulfate as neuroprotection in acute stroke. N Engl J Med.

[23] Toffa DH, Magnerou MA, Kassab A (2019). Can magnesium reduce central neurodegeneration in Alzheimer’s disease? Basic evidences and research needs. Neurochem Int.

[24] Goto Y, Nakamura M, Abe S (1993). Physiological correlates of abnormal behaviors in magnesium-deficient rats. Epilepsy Res.

[25] Weisinger JR, Bellorín-Font E (1998). Magnesium and phosphorus. Lancet.

[26] Moe SM (2008). Disorders involving calcium, phosphorus, and magnesium. Prim Care—Clin Off Pract.

